# The research infrastructure of Chinese foundations, a database for Chinese civil society studies

**DOI:** 10.1038/sdata.2017.94

**Published:** 2017-07-25

**Authors:** Ji Ma, Qun Wang, Chao Dong, Huafang Li

**Affiliations:** 1 Indiana University Indianapolis, Lilly Family School of Philanthropy, Indiana 46202, USA; 2 Intetix Institute, Beijing 100020, PR China; 3 Indiana University Bloomington, School of Public and Environmental Affairs, Indiana 47405, USA; 4 CDD Tech Ltd., Beijing 100102, PR China; 5 Grand Valley State University, School of Public, Nonprofit and Health Administration, Michigan 49504, USA

**Keywords:** Politics, Society, Sociology

## Abstract

This paper provides technical details and user guidance on the Research Infrastructure of Chinese Foundations (RICF), a database of Chinese foundations, civil society, and social development in general. The structure of the RICF is deliberately designed and normalized according to the Three Normal Forms. The database schema consists of three major themes: foundations’ basic organizational profile (i.e., *basic profile*, *board member*, *supervisor*, *staff*, and *related party* tables), program information (i.e., *program information*, *major program*, *program relationship*, and *major recipient* tables), and financial information (i.e., *financial position*, *financial activities*, *cash flow*, *activity overview*, and *large donation* tables). The RICF’s data quality can be measured by four criteria: data source reputation and credibility, completeness, accuracy, and timeliness. Data records are properly versioned, allowing verification and replication for research purposes.

## Background & Summary

Scholarly interest in civil society in contemporary China began in the mid-1980s, especially after the 1989 Tiananmen Incident^1^. Studies on Chinese civil society have various theoretical and practical implications, e.g., the state-society relationship and the democratization process in China. However, although China is becoming an important and rapidly growing political and economic power, our knowledge about Chinese civil society remains limited. The majority of previous studies on Chinese civil society are dominated by paradigms originating in Western political philosophy or the so-called ‘Anglosphere’ cultures^2^, e.g., the Tocquevillian civil society paradigm, which regards civil society as a necessary power to check the state, or the ‘state-corporatism’ paradigm, which considers civil society as a dependency of the state^3^. However, none of these paradigms can provide sufficient explanations for understanding Chinese civil society. The lack of cultural diversity and indigenous paradigms is a major challenge for studying civil society in China^4^, but little progress has been made since the 1980s^5,6^.

A major challenge for progress in the study of civil society in China is the lack of data for empirical studies on which new paradigms can be built and tested. In the United States, data extracted from Internal Revenue Service (IRS) 990 Forms (Form 990, Form 990-EZ, Form 990-PF, and Form 990-N) has boosted knowledge production on civil society and the non-profit sector. However, unlike the United States, where there are numerous institutions that provide database services to scholars (e.g., GuideStar, Urban Institute, and Foundation Center *et al.*), few counterparts in China have emerged and none of them can adequately serve academic research—the datasets are neither structured for research purposes nor easily accessible.

In responding to this critical data scarcity challenge, we built a database for studying Chinese foundations—the Research Infrastructure of Chinese Foundations (RICF). The foundation (*jijinhui*) is one of the three organizational forms of registered NGOs. The other two are membership-based association (*shehui tuanti*) and social service organization (*shehui fuwu jigou,* formerly named as *minban feiqiye danwei*). Among these three organizational forms, foundations are the most developed organizational form and dominant civic power in China, and they are critical for strategically preserving the autonomy of civil society from state control^7^. Empirical studies about Chinese foundations can generate important theoretical and practical implications for Chinese non-governmental organizations and civil society. For example, the board interlock analysis using RICF discovers the contingent relationship between state power and business elites, and this relationship provides empirical evidence for a new paradigm of ‘networked civil society’ within which networked multipolar groups share power and achieve an equilibrium rather than behaving independently^7^. A critical discourse analysis using RICF reveals that the Chinese government tends to co-opt foundations formed by firms and entrepreneurs. These foundations can generate sufficient funding from their founding firms and entrepreneurs. However, the government tends to restrict the activities of foundations that use diverse revenue strategies^8^. This differentiated control mechanism challenges the dominant ‘conflicting paradigm’ (i.e., state power always conflicts with civic power) in the Western world^9^. A multilevel analysis using RICF suggests that the distribution of resources is highly imbalanced among foundations and that some types of foundations are more capable of mobilizing resources^10^.

This paper intends to help scholars understand and make the best use of RICF. It introduces the database structure, how to validate the data, the data collection procedure, and the data quality control mechanism.

## Methods

**Figure 1:**
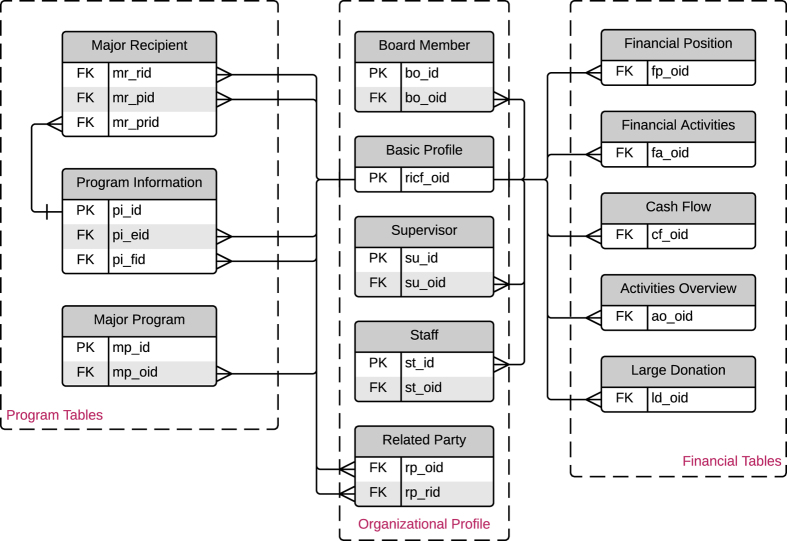
Database Schema. Only primary keys and foreign keys are listed in the Schema; refer to the codebook for complete data fields. PK=Primary Key; FK=Foreign Key.

The database structure of RICF is designed and normalized by adhering to the Three Normal Forms (3NFs)—a series of rules for organizing the attributes within a table and the relationships between different tables^11^. As Fig. 1 illustrates, the database schema consists of three major themes: a basic organizational profile of foundations (i.e., *basic profile*, *board member*, *supervisor*, *staff*, and *related party* tables), program information (i.e., *program information*, *major program*, *program relationship*, and *major recipient* tables), and financial information (i.e., *financial position*, *financial activities*, *cash flow*, *activity overview*, and *large donation* tables). The primary key (PK) in each table is a unique identifier, and the foreign key (FK) is used to establish connections between different tables. For example, the ricf_oid in the *basic profile* table is a PK that records the organizations’ unique IDs, but in the *program information* table, it is an FK to link back to the *basic profile* table; therefore, while analyzing programs, scholars can use this data field to retrieve the organization’s profile.

The data are crawled, parsed, and compiled manually or automatically by computer programs (Python Scrapy and other data processing packages, e.g., Pandas) from the following six sources, which are ranked by their credibility:

1. Annual reports and audited financial reports. Chinese foundations are required to submit their annual reports to the civil affairs departments with which they are registered. These reports can be obtained from the foundations’ or the government’s official websites. The addresses of foundations’ official websites are recorded under ba_wb in the *basic profile* table.
2. Information disclosed by supervising government departments. For example, annual filing disclosed by the Civil Organization Administration Bureau of the Ministry of Civil Affairs (http://jjh.chinanpo.gov.cn) and the Shanghai Administration Bureau of NGOs (http://xxgk.shstj.gov.cn/), among others. The Ministry of Civil Affairs (http://www.mca.gov.cn/) has a list of websites of supervising government departments.
3. Information disclosed by the China Foundation Database (http://chinafoundation.org.cn; an information-disclosing platform supervised by the Civil Organization Administration Bureau, closed in early 2016 for unknown reasons).
4. Information disclosed by the China Foundation Center (http://foundationcenter.org.cn; an information-disclosing platform run by a nonprofit organization).
5. News from the foundation’s official website. The website snapshots are taken and stored under the ‘raw data’ folder (see Data Records section below; the same for source #6).
6. News from credible magazines or websites.

### Code availability

The raw data are processed using Python 2.x. For users’ convenience, we geocode the foundation’s address using Python geocoding package Geocoder (https://geocoder.readthedocs.io) and following two settings: 1) ArcGIS is preferred because of precision, and 2) the addresses not successfully geocoded by ArcGIS are recoded by Google GIS. Codes for geocoding are available at https://github.com/ma47/RICF.

## Data Records

The development version of the data is available at GitHub (https://github.com/ma47/RICF). Under the root repository, we named and organized folders and files as follows:

1. Foundation data records are organized by year (e.g., folders named ‘2013’ and ‘2014’). Each file represents a table in the database schema (Fig. 1). The data files are tab-delimited and use UTF-8 encoding.
2. ‘codes’: this folder contains codes for particular purposes, e.g., codes for geocoding.
3. ‘raw data’: this folder contains raw materials from which the data are extracted, e.g., annual reports and website snapshots.
4. ‘RICF codebook.xlsx’: Codebook in MS Excel format.
5. ‘how to cite.bib’: Citation information of RICF.
6. ‘README.md’: General instructions.

All revisions are properly logged using GitHub’s version control function. Users can easily track the changes or revert to a specific version. Once we start to release the data tables of a specific year (e.g., 2013), a stable version is published on GitHub (https://github.com/ma47/RICF/releases) and updated on Harvard Dataverse (Data Citation 1; files are tagged with version names described below). The stable version contains all the repositories and files except the ‘raw data’ and ‘codes’ folders.

The version name is formatted as ‘v.Year.MajorRevision.MinorRevision’ for the purpose of version control. The *Year* field indicates the year for which the most recent records are available. For example, ‘2014’ means that the most recent records in this release are from 2014 and that this version also contains earlier records that date back to 2013 (current first release; we are scheduled to release the data dating back to as early as 2008 and will put this change in the revision history). The *MajorRevision* field is updated when new data tables are added to the package. In doing so, we can strike a balance between the timeliness of research and the accuracy of data. First, if we release a stable version only when all the data tables of a year are ready, it will not satisfy timely reasearch demands. Second, most of the time, scholars use only a proportion of the data tables. Therefore, releasing stable versions table by table instead of year by year should achieve a better balance between the timeliness of research and the accuracy of data. The *MinorRevision* field is updated when erroneous records are corrected.

## Technical Validation

### Data quality dimensions

Data quality is usually defined as ‘fitness for use by data consumers’^12^ and relates not only to the content of data but also to the way that data are utilized and whether data consumers are satisfied with using data for their purposes. The diverse nature of data quality results in many data quality dimensions derived from different needs.

Four typical dimensions have significant impacts on the goal of RICF: data source reputation and credibility, completeness, accuracy, and timeliness^13^. This section introduces how these four dimensions are employed to measure the extent to which RICF is reliable, complete, accurate, and timely.

### Data source reputation and credibility

Data source reputation refers to whether the data source is in high standing; credibility is the degree to which the data are considered true and credible to data consumers^12,14^. The combination of reputation and credibility indicates whether the data can be trusted and represents the way in which the data source convinces data consumers that the data are considered to be true and credible^12^.

The RICF data are collected from the six different sources listed above. These sources are ranked by their reputation and credibility. When conflicts occur, the rankings will be used for the evaluation of accuracy. For instance, when a piece of information about an organization from Rank 2 contradicts the same information from Rank 1, RICF uses information from Rank 1 rather than that from lower ranks.

### Completeness

The completeness of data in RICF is defined as ‘the extent to which data are of sufficient breadth, depth, and scope for the task at hand,’^15^ or ‘the quotient of the number of non-null values in a source and the size of the universal relation’^14^. The universal relation is that consisting of all attributes of the global schema. RICF considers three types of completeness in the design process:

1. *Schema completeness* refers to the degree to which the profiles of a source (e.g., entities and attributes) are not missing from the database schema. This type of completeness is controlled and can be evaluated by the Database Schema of the RICF (Fig. 1).
2. *Column completeness* measures the integrity of columns in a table. It is also known as attribute completeness in the relational database. This type of completeness is controlled by the RICF codebook.
3. *Population completeness* measures the integrity of observations compared to a reference population. Table 1 provides two other data sources for evaluating the RICF’s population completeness.

A major resource for determining and improving the schema and column completeness is the Chinese foundations’ annual reports. The Regulations on the Management of Foundations^16^ requires all foundations to submit annual reports to the civil affairs departments with which they are registered. The annual reports contain three main types of information:

1. Organizational and operational profiles, including personnel, board of directors, board of supervisors, annual evaluation results, tax exemption status, etc.
2. Financial information, such as assets, donation income, and expenses, etc. The financial information should have been audited by a qualified accounting firm before submission.
3. Project summaries that report the focuses of projects, beneficiaries, and funding received and spent, etc.

### Accuracy

Accuracy refers to the closeness of a value to another value that is considered correct^17^. Regarding accuracy, a data value must be correct and stored in a proper form (e.g., consistent and unambiguous); therefore, both the content of data and form of storage are indispensable for accuracy^18^. RICF uses three methods to ensure data accuracy:

1. Triangulation using data from different sources. All the source files used in compiling the data are retained for reference.
2. Ranking priorities for reputation and credibility of the data sources discussed in the previous section.
3. Normalization using 3NF rules to maintain the integrity and consistency of the stored data.

### Timeliness

Timeliness measures the extent to which the data are sufficiently timely. Two concepts are important for timeliness: currency and volatility. Currency is defined as ‘the age of the data when it is delivered to users; volatility refers to ‘the length of time during which the data remain valid’^19^. For instance, a grocery store may need to update the transaction data daily to generate a timely sales report and provide critical information for inventory.

Timeliness is highly dependent on the scenarios in which the data will be used. Most of the data in RICF are static data, i.e., data that will not be updated during their lifetime (e.g., name of the foundation and registration number, etc.) and seldom-updated data (annual income and expenses, etc.). The volatility is long, and for our research purposes, the currency does not need to be as short as daily or monthly. Therefore, the RICF has a comprehensive update scheduled once a year, and the currency is set as one year. For example, the 2015 annual data of most foundations were released and available to us around August 2016 (i.e., data became available on foundations’ websites or government’s websites), and RICF then will compile and release these data one year later, i.e., around August 2017.

We believe that, at this stage, the four-dimension evaluations—data source reputation and credibility, completeness, accuracy, and timeliness—can effectively serve the research interests of Chinese foundations and Chinese civil society in general.

### Null values

Another important issue is how to address null values, which usually indicate missing values; however, it is important to understand the reasons for missing values because it is relevant to the evaluation of completeness. A value may be missing on three occasions: (1) the value does not exist; (2) the value exists but is unavailable; and (3) it is unknown whether the value exists or not^20^. The word ‘exist’ is defined here from an ontological perspective. Whether a value exists is not judged by the availability of data but rather by reasoning. While developing the codebook according to the rule of column completeness, all of the foundations are expected to have values for all the variables. Therefore, conditions 1 and 3 are not applicable to RICF. All the null values fall under condition 2.

### Validation experiments

We did two experiments to test the validity of the data: the descriptive and regression experiments.

#### Descriptive experiment

We calculated the descriptive statistics of selected varibles using one of the data sources and compared the results with RICF (Table 2). The 95% coefficent intervals suggest that the distributions of these varibles, although from different sources, are largely overlapped.

#### Regression experiments

In one of our empirical studies, we hand-coded one of the variables, i.e., the number of government officials on foundations’ boards^7^. The regression results using RICF and hand-coded dataset are congrent with each other.

## Usage Notes

Users may face the encoding problem of Chinese characters. All the records use UTF-8 and are tab-separated. Please pay special attention while importing files.

## Additional Information

**How to cite this article**: Ma, J. *et al.* The research infrastructure of Chinese foundations, a database for Chinese civil society studies. *Sci. Data* 4:170094 doi: 10.1038/sdata.2017.94 (2017).

**Publisher**’**s note**: Springer Nature remains neutral with regard to jurisdictional claims in published maps and institutional affiliations.

## Supplementary Material



## Figures and Tables

**Table 1:** Number of foundations from three sources: the RICF, China Statistical Yearbook (Yearbook) and China Foundation Center (CFC).

Year	RICF	Yearbook	CFC	Year	RICF	Yearbook	CFC
1981	5	NA	8	1998	535	NA	515
1982	13	NA	15	1999	545	NA	526
1983	22	NA	16	2000	552	NA	539
1984	30	NA	22	2001	568	NA	554
1985	40	NA	36	2002	587	NA	572
1986	59	NA	54	2003	614	954	597
1987	68	NA	66	2004	695	892	745
1988	96	NA	94	2005	832	975	891
1989	140	NA	140	2006	982	1,144	1,056
1990	161	NA	162	2007	1,188	1,340	1,280
1991	184	NA	185	2008	1,416	1,597	1,533
1992	264	NA	261	2009	1,665	1,843	1,826
1993	325	NA	320	2010	2,040	2,200	2,213
1994	394	NA	388	2011	2,430	2,614	2,608
1995	463	NA	453	2012	2,880	3,029	3,009
1996	502	NA	490	2013	3,344	3,549	3,627
1997	525	NA	506	2014	4,233	4,117	4,211
Sources: China Statistical Yearbook 2015 (ref. 21), The 2014 Statistical Report of Social Service Development^22^ and The CFC Independent Research Report^23^.							

**Table 2:** Validation data using different sources.

	**Total Income**		**Total Donation Income**		**Total Expense**		**Net Asset**	
	**RICF**	**CFC**	**RICF**	**CFC**	**RICF**	**CFC**	**RICF**	**CFC**
N	2,762	3,136	2,762	3,136	2,762	3,124	2,907	3,134
Mean	1.39	1.30	1.15	1.08	1.01	0.94	3.15	3.01
s.d.	7.21	6.80	6.22	5.87	6.25	5.90	13.45	12.99
95% CI Upper	1.66	1.54	1.38	1.28	1.24	1.15	3.64	3.46
95% CI Lower	1.12	1.06	0.92	0.87	0.78	0.74	2.66	2.55
Note: All numbers except for sample size (N) are in ten million Chinese Yuan (CNY). CFC, China Foundation Center; RICF, Research Infrastructure of Chinese Foundations.								
